# Possibility to extend the shelf life of NFC tomato juice using cold atmospheric pressure plasma

**DOI:** 10.1038/s41598-020-77977-0

**Published:** 2020-12-01

**Authors:** Agnieszka Starek, Agnieszka Sagan, Dariusz Andrejko, Barbara Chudzik, Zbigniew Kobus, Michał Kwiatkowski, Piotr Terebun, Joanna Pawłat

**Affiliations:** 1 Department of Biological Bases of Food and Feed Technologies, University of Life Sciences in Lublin, Głęboka 28 st, 20-612 Lublin, Poland; 2 Department of Biological and Environmental Education with Zoological Museum, Maria Curie-Skłodowska University, Akademicka 19 st, 20-033 Lublin, Poland; 3 Department of Technology Fundamentals, University of Life Sciences in Lublin, Głęboka 28 st, 20-612 Lublin, Poland; 4 Institiute of Electrical Engineering, Electrotechnologies, Lublin University of Technology, Nadbystrzycka 38a st, 20-618 Lublin, Poland

**Keywords:** Microbiology, Nutrition, Chemical safety, Applied physics

## Abstract

Cold Atmospheric pressure Plasma (CAP) is a non-thermal method used in food processing. CAP generated with the use of nitrogen in a Glide-arc device for 300 to 600 s exhibited high potential for microbial decontamination and did not induce substantial changes in the physicochemical properties of NFC tomato juice. Samples exposed to cold atmospheric plasma had mostly an intact structure, as revealed by digital microscopy. The investigations indicate that CAP can be applied for biological and chemical waste-free decontamination of food and extension of its shelf life.

## Introduction

At present, not-from-concentrate juices (NFC) also referred to as one-day or fresh juices are premium products on the food market. They are produced by pressing fruit pulp or mechanical mashing raw materials. Many scientific studies confirm that cloudy juices (vs. clear juices) contain several-fold higher amounts of beneficial bioactive compounds, which exhibit strong anti-cancer, anti-inflammatory, and anti-aging activities. One of such products, i.e. tomato juice, which is one of the most widely produced juices worldwide, deserves special attention. The high consumption of this product is associated with not only its refreshing taste stimulating appetite but also its high nutritional value. Tomato juice is characterized by low calorific value, richness of minerals, and high content of nutritionally valuable carotenoids, flavonoids, and vitamin C^1–6^.

Unfortunately, since no heat treatment is applied, NFC juices must be transported and stored in refrigerated conditions (2–6 °C) and their shelf life is only one day. A serious problem for the industry is the microbiological instability of such products during longer storage associated with not only adverse organoleptic and sensory changes but also, most importantly, microbiological hazards to the consumer. Due to the low degree of processing, unpasteurized juices are a reservoir of natural microflora, but may also be vectors of undesirable pathogenic microorganisms^2,7–10^.

Therefore, it is important to search for new methods for processing freshly pressed fruit or vegetable juices yielding a microbiologically pure product with an extended shelf life and all nutritional values indispensable for the proper function of the consumer's organism.

Cold atmospheric plasma (CAP) is an interesting alternative to the current food processing methods^11–15^. The suitability of this technique is supported by its high efficiency in removal of microorganisms without the need for thermal treatment of the raw material. CAP is a unique state of matter with properties of ionized gas, in which many biochemically active factors like charged particles (ions and electrons), photons and ultra-violet (UV) radiation, free radicals, reactive oxygen and nitrogen species (RONS), including atomic nitrogen (N), and nitric oxide (NO), peroxynitrite (ONOO^−^) and derived species, atomic oxygen (O), hydroxyl radical (^•^OH), superoxide (O_2_^−^), hydrogen peroxide (H_2_O_2_), etc. can be generated, both in surrounding gaseous environments, and as a result of plasma interaction with liquid media^16,17^. Non-equilibrium low pressure-plasmas are extensively studied as a new sterilization and decontamination method in the field of medicine, sanitation and food processing^18–21^. The use of cold atmospheric plasmas as a sterilization technique has many advantages, including low costs, avoidance of chemicals and undesirable changes in products associated with heat treatment. Studies on the effect of plasma on microbial cells in organic media have shown that CAP induce significant oxidative stress in bacterial and fungal cells and trigger intracellular production of reactive oxygen species (ROS). The plasma-generated reactive species and plasma induced endogenous ROS possibly contributed to microbial death synergistically^22^. These oxidative stresses can cause cell death by damaging macromolecules such as deoxyribonucleic acid, proteins, lipids, and affecting the cellular metabolism^23–26^.

Available research results have indicated the antibacterial and antifungal activity of cold atmospheric plasma applied to pasteurized juices contaminated with pathogenic microflora prior to the treatment. Some authors have analyzed the impact of this technique on the physicochemical properties of this type of products. For example Xu et al*.*^22^ conclude that high voltage atmospheric cold plasma (HVACP) treatment can be an effective nonthermal technology to control, or potentially eliminate, *Salmonella* in pasteurized orange juice with minimal quality alteration (color, ^o^Brix, pH). It was shown that 120-s HVACP direct treatment in air reduced vitamin C by 22% (compared to 50% for heat pasteurization). In Liao et al*.*^27^ study, a dielectric barrier discharge (DBD)‐CAP exhibited strong bactericidal effect on *Escherichia coli* in apple juice, reducing number of colony forming units- CFU/ml by 3.98 to 4.34 log. In addition, the CAP exposure had slight effect on the °Brix, pH, titratable acidity, color values, total phenolic content, and antioxidant capacity of this product. Dasan et al*.*^28^ observed reduction of the bacterial count in orange, apple, and tomato juices. Xiang et al.^29^ inactivated *Zygosaccharomyces rouxii*, while Surowsky et al*.*^30^ reported inactivation of *Citrobacter freundii* in apple juice using CAP. Pankaj et al*.*^31^ observed reduced *Sacharomyces cerevisiae* counts in white grape juice. Exposition of raw vegetable juice to plasma treatment can lead to the increase of polyphenols and flavonoids but overexposure to plasma have a tendency to degrade the bioactive compounds, thus the selection of suitable process conditions should be considered in the design and performance of this treatment. When a substrate gas is passing via electrical discharge zone, a number of radicals and singlets are formed, which could also react with fructose and glucose^32^. This is confirmed by the analyzes of Almeida et al*.*^33^ who observed a reduction of 12 and 22% in oligosaccharide, fructose and glucose when applying direct atmospheric cold plasma to orange juice.

The aim of the present work was to examine the impact of CAP treatment on the different quality parameters of NFC tomato juice (microbial quality, physicochemical and structural properties). The CAP usefulness in prolonging the shelf-life of fresh tomato juice with minimized losses of valuable nutrients was also evaluated.

## Materials and methods

### Preparation of tomato juice

Fresh tomatoes (*Lycopersicon esculentum* cv. Apis F_1_) were purchased from the Horticultural Farm, where biological protection measures are applied (Lublin Province, Poland). The tomatoes were washed by rinsing with tap water and dried with paper towels. Next, juice was made using a Sana EUJ-707 single-screw press, Omega Products, South Korea (the skin and seeds were removed automatically). The juice was divided into six different parts as a control sample and test samples for the atmospheric plasma treatment. Samples of the juice were used for microbiological, physicochemical and microstructure analysis.

### Plasma treatment system

**Figure 1:**
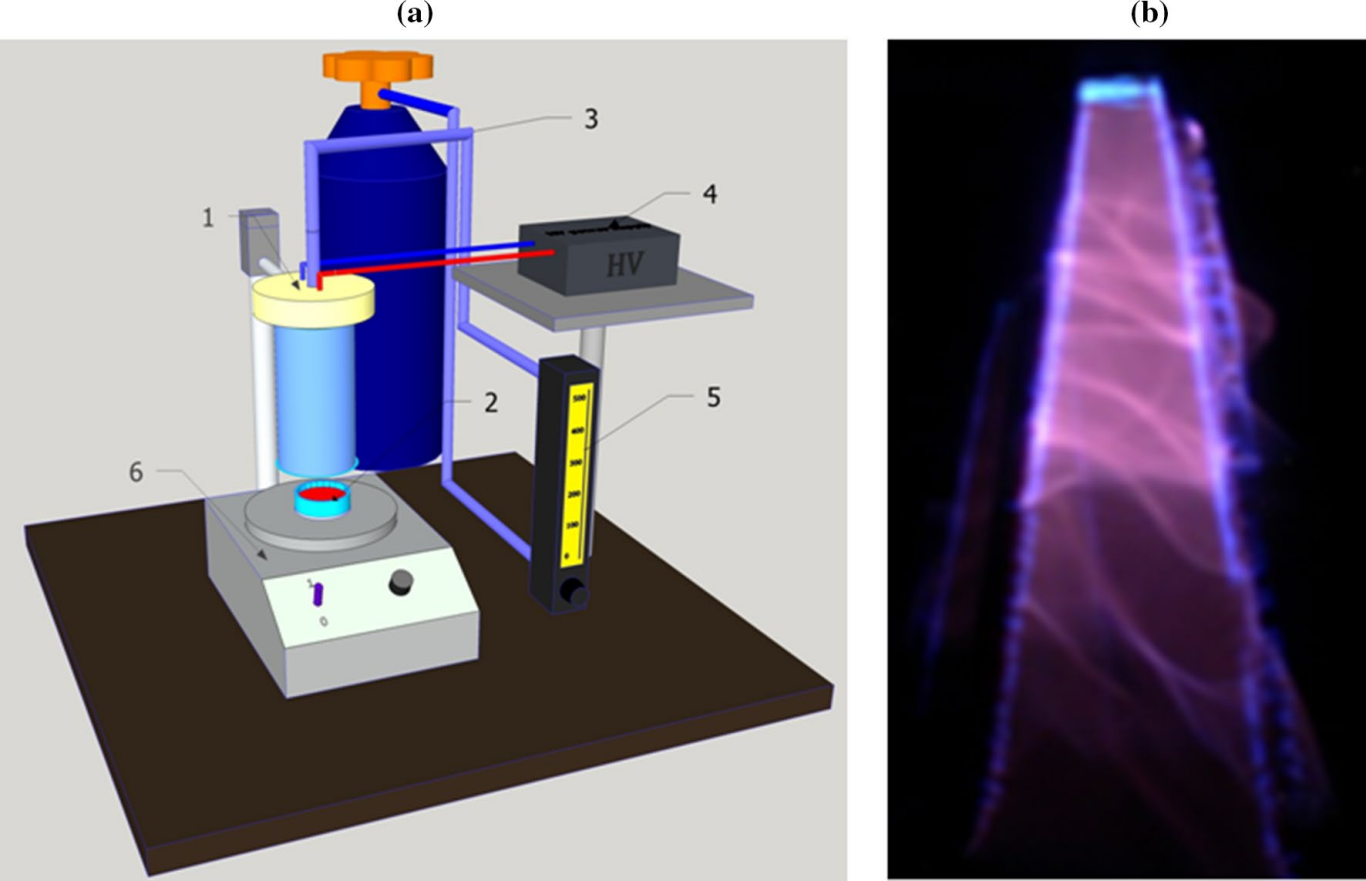
**(a)** Schematic diagram of the atmospheric plasma treatment system (1—GAD, 2—sample, 3—gas supply, 4—power supply, 5—flowmeter, 6—magnetic stirrer); **(b)** electrical discharge in GAD reactor supplied with nitrogen gas.

Fresh tomato juice was subjected to cold plasma generated with the use of a Glide-arc type plasma reactor operating at atmospheric pressure. Two 10-cm copper wire electrodes were mounted in the reactor. The system of process gas distribution allowed targeting the gas stream along the electrodes, which enabled the generated electric arc to move along the electrodes with a forced gas flow, thus increasing the discharge volume. The schema of the juice treatment set-up and electrical discharge photo of GAD reactor are depicted in Fig. 1.

Nitrogen (purity 6.0, Linde Gas Poland) with a flow of 440 l/h was supplied to the Glide-arc type reactor (50 Hz, 3.8 kV peak voltage, 40 W). The gas stream leaving the reactor was targeted at the tomato juice (50 ml) with an input temperature of 25 °C, which was placed on the feeder for the fixed time (30, 60, 120, 300, and 600 s). Six samples of 50 ml tomato juice were prepared in duplicate, and five from them were plasma treated for 0, 30, 60, 120, 300 or 600 s. During the plasma treatment the juice samples were placed in an open batch type glass container with a diameter of 6 cm and walls with a height of 4 cm. The container was set on a magnetic stirrer in an ice bath. During plasma treatment, the juice samples were stirred at 50 rpm. Flow rate of supply gas was quite high so the prevailing active species originated from nitrogen; however, some gas mixing with the ambient air also could take place. After the longest treatment, the temperature of the juice increased only slightly to a maximum of 29 °C.

### Microbiological analysis

To assess the effectiveness of the CAP treatment in microbiological decontamination, the juice samples after CAP treatment were stored in sterile containers in refrigerator for 1, 4, 7, and 10 days. After this time, the containers with juice were transported in refrigeration conditions to an accredited microbiological food processing laboratory (Polish Centre for Accreditation AB 444). In the laboratory, the juice samples were serially diluted and inoculated on suitable culture solid media to determine the total number of aerobic microorganisms^34^, lactic acid bacteria^35^, coliform bacteria^36^, yeasts^37^. From the first samples repetition there were made three independent microbiological cultures and colony counts, while from the duplicate samples two determinations were performed (*n* = *5* for each treatment time).

### Physicochemical analysis

#### Determination of pH

The juice pH (10 mL) was measured at a temperature of 20 ± 1 °C using 780 pH meter, Metrohm, Herisau, Switzerland. Before and after all measurements, the electrode tips were thoroughly rinsed with distilled water. The pH meter was calibrated with standard buffer solutions which had pH values in the range 4–9.

#### Determination of total soluble solid content (°Brix)

°Brix of sample was measured at 20 ± 1 °C using a refractometer PAL-1 Atago, Tokio, Japan^42,43^. The prism of refractometer was cleaned with distilled water after each analysis.

#### Determination of total carotenoids content

Total carotenoid content was determined by a modified method proposed by Gonzales-Casado et al*.*^38^ using a spectrophotometer Thermo Scientific UV–Vis Helios Omega 3, Waltham, Massachusetts, USA. The absorbance of the organic phase for the total carotenoid content was measured in triplicates at 470 nm versus a blank of hexane.

#### Determination of ascorbic acid

The content of vitamin C was determined with the Tillmans method. This titration method is based on reaction of ascorbic acid with a 2,6-dichlorophenolindophenol solution (Tillmans reagent). 15 g of juice was transferred to 100 cm^3^ volume container, which was further filled with 2% oxalic acid solution, mixed and put in the darkness for 15 min. Then, the content was filtered. For the final measurements, 25 cm^3^ of filtrate was put in 50 cm^3^ container, which was subsequently filled with 2% oxalic acid solution, mixed and titrated with Tillmans reagent until a pink color was visible. The volume of the reagent (cm^3^) required for oxidation of 1 mg of ascorbic acid was assumed as the volume of 2,6-dichlorophenolindophenol titer^39^.

### Microscopic analysis

Samples of freshly pressed juice and juice treated with cold plasma for 30, 60, 120, 300, and 600 s were analyzed with an optical microscope KEYENCE VHX 950F (Japan) equipped with a digital camera to evaluate the degree of destruction of storage parenchyma cells.

### Statistical analysis

The obtained results were statistically analyzed with Statistica software^40^ via analysis of variance (ANOVA). The significance of differences between the evaluated mean values (in figures) were analyzed with the Tukey test at a significance level of p < 0.05. On the basis of results from experiments with individual samples mean values and whiskers representing standard deviations were calculated. The results of the microbiological analyses and physicochemical properties are presented as average values of five and three measurements in each sample, respectively.

## Results and discussion

### Microbiological analysis

The number of microorganisms per volume unit in the juice indicates its microbiological quality and suitability for consumption. Detection of even minimal numbers of pathogenic bacteria such as *Listeria monocytogenes* or *Salmonella* makes the product unfit for consumption and necessitates withdrawal of an entire batch. Additionally, the presence of *Escherichia coli* must be under strict control and should not exceed 100 CFU/g (2 log_10_ CFU/g). An increased count of these fecal bacteria indicates contamination during the production process and disqualifies the food product.

No pathogenic *Listeria monocytogenes* and *Salmonella* bacteria were detected in the control and plasma-treated tomato juice samples. *Escherichia coli* were below the limit of quantification by the culture methods (< 10 CFU/g) as well. The presence of mesophilic aerobic microorganisms, e.g. lactic acid bacteria and yeasts, in unpasteurized vegetable juice is a natural phenomenon associated with the presence of these microorganisms on vegetables and utensils used for pressing the juice. These microorganisms are responsible for spoilage and fermentation of stored juice, making it unfit for consumption within one to several days.

The amended Polish Regulation of the Minister of Health of January 13, 2003 (on the maximum levels of chemical and biological impurities allowed to be present in food and food ingredients, approved additives, processing aids, or on the surface of foods) specifies the microbiological quality only for pasteurized fruit and vegetable juices^41^. According to the Regulation, the allowable count of mesophilic aerobic microorganisms in pasteurized juice should be equal to or lower than 10^3^ CFU/g (3.0 log_10_ CFU/g) and can be in the range of 10^3^–10^4^ CFU g (3.0–4.0 log_10_ CFU/g) in maximum two of five samples collected from a batch. An amount exceeding 10^4^ CFU/g disqualifies the entire batch of the product. The same Regulation specifies the allowable amount of yeast and mold in pasteurized juices at a level equal to or below 10 CFU/g (1 log_10_ CFU/g) and in the range of 10—2 × 10 CFU/g in two of five samples.

No legal regulations define the allowable count of mesophilic aerobic microorganisms in fresh unpasteurized NFC juices. It is only indicated that detection of any amount of pathogenic bacteria such as *Salmonella* and *Listeria monocytogenes* disqualifies the product. The European Commission Regulation No. 2073/2005 of November 15, 2005 on microbiological criteria for foodstuffs^42^ establishes the allowable contamination with *Escherichia coli* in fresh unpasteurized fruit and vegetable juices at or below 100 CFU/g (≤ 2 log_10_ CFU/g) and in the range of 100–1000 CFU/g (2–3 log_10_ CFU/g) in two of five samples. There is no information in the literature on the permissible number of aerobic microorganisms in unpasteurized tomato juice. It is generally assumed that the presence of > 4 log10 CFU/g of microorganisms is associated with rapid deterioration of juice and its unfitness for consumption.

**Figure 2:**
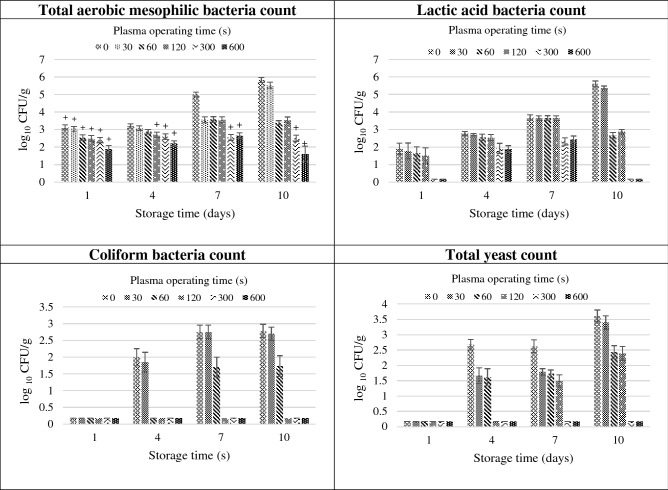
Effect of the duration of the CAP treatment on the microbiological quality of NFC cold-stored tomato juice; based on the available legal regulations for pasteurized juices: “ + ” above the bar in the graph of the total number of aerobic microorganisms means full consumption fitness of the product. Average values (*n* = *5*) with standard deviation are provided.

The results of the microbiological assays applied in the fresh tomato juice exposed to CAP and stored in refrigeration conditions are shown in Fig. 2.

On the first day after the preparation, the control (plasma untreated) juice samples exhibited a relatively high number of mesophilic aerobic microorganisms, i.e. at an average level of 3.1 log_10_ CFU/g. A similar total count of aerobic microorganisms (3.2 log_10_ CFU/g) was detected after 4 days of cold storage of the juice samples. On the consecutive days of storage, this number increased significantly due to the microbial multiplication to the level of 5.0 log_10_ CFU/g after 7 days and 5.8 log_10_ CFU/g after 10 days of storage. On day 1, the control juice samples exhibited the presence of lactic acid bacteria at a level of 1.9 log_10_ CFU/g. Their number substantially increased on the subsequent storage days to reach 2.8 log_10_ CFU/g, 3.7 log_10_ CFU/g, and 5.6 log_10_ CFU/g after storage days 4, 7, and 10, respectively. Coliform bacteria were present at a very low level of 0.6 log_10_ CFU/g on the first day, but they multiplied intensively throughout the storage time and reached 1.2 log_10_ CFU/g, 2.7 log_10_ CFU/g, and 2.8 log_10_ CFU/g after 4, 7, and 10 days, respectively. Similarly, the small number of yeast detected on the first day, i.e. 0.6 log_10_ CFU/g, increased to 2.6 log10 CFU/g after 4 and 7 days of storage and 3.6 log_10_ CFU/g after 10 days. In accordance with the legal regulations on the microbiological quality of pasteurized juices, the results of the microbiological cultures indicate that the control tomato juice met the criteria for consumption on the first day after pressing. After 4 days of refrigerated storage, the total number of aerobic microorganisms was also within the shelf-life criteria, but the yeast content increased excessively. The longer storage time (7 and 10 days) was associated with further growth of the microorganisms, which made the juice unfit for consumption.

After 10 days of storage, the number of CFU/g for the total mesophilic bacteria in the control juice samples and in the samples treated with plasma for the shortest time (30 s) increased by about 2.5 log_10_. Similar results were obtained for lactic acid bacteria, for which the number of CFU/g increased by over 3 log_10_ after 10 days of storage. Such a result is not surprising because some strains of bacteria, capable to proliferate in the cooling conditions, multiplied at the expense of the nutrients contained in the juice. The products of metabolism of these bacteria spoiled the juice and made it unfit for consumption. The median time of plasma treatment (60 and 120 s) caused that, after 10 days of storage, the numbers of CFU/g of total mesophilic bacteria and lactic acid bacteria were moderately increased in comparison to the samples taken at the first day. After the longest and the second-longest time of plasma treatment after 10 days of storage the number of total mesophilic bacteria CFU/g was at the same level as at the first day. The possible explanation of this phenomenon is that the long plasma treatment caused eradication of this species of bacteria, which are capable to proliferate in low temperature. Different bacteria species and even strains probably differ in their sensitivity to the toxic effects of active molecules generated by the plasma. Different results were obtained for lactic acid bacteria in the samples treated for 300 and 600 s, where at the first day the number of CFU/g was very low, than increased on the days 4–7, while after 10 days of storage decreased almost to zero. It can certainly be said that the lactic acid bacteria turned out to be very sensitive to the long exposure to plasma and on the first day after treatment their level was very low in comparison to the control. After a few days of storage, there was observed a slight recovery of these bacteria and regaining the ability to proliferate, but after a longer storage in refrigerated conditions, they did not find suitable conditions for survival there. Explanation the mechanisms of the observed microbial recovery or death after plasma treatment would require further studies at a biochemical and molecular level. These issues are poorly studied and are certainly an interesting field of research for the future. Our results show that susceptibility of diverse microorganisms to plasma treatment differ depending on time of treatment. It was shown that the coliform bacteria are very susceptible, because their number of CFU/g was at a very low level in samples treated for 120 s or longer, for the whole studied storage period. On the other hand, yeasts after the same time of treatment recovered and proliferated during storage. Yeasts were eradicated only by the longest and second-longest plasma operating time. Nevertheless, the obtained results are promising and give hope for the use of plasma for extending the shelf life of unpasteurized juices.

Another very important factor affecting microbial inactivation is time- dependent chemistry of RONS in the solution^16,43^. Short-lived RONS primarily formed in plasma are delivered with exhaust gas to juice, then products of their reactions can last for relatively long time or further take part follow-up reactions also affecting the behavior of microorganisms.

The radical species emitted by a gas–plasma operation are mainly reactive nitrogen species, like nitric oxide (NO) and nitrogen dioxide (NO_2_), as well as reactive oxygen species, like ozone (O_3_), hydroxyl radicals (OH^.^), superoxide (O2 ^−^), and singlet oxygen (_1_O_2_). Plasma treatment of an organic medium, like a juice, induces the increase of reactive oxygen and nitrogen specie (RONS) concentration in the medium. These environmental stresses can potentially trigger cell degradation by damaging bio-macromolecules such as proteins, lipids, and DNAs, thereby affecting the cellular metabolism, electron transportation, intracellular electric fields generation, and intracellular ROS production. The pH of the liquid has significant effects on the concentration of O_2_^−^, HO_2_, O_2_NO^−^, and O_2_NOH generated during plasma operating.

The increasing concentration and the higher permittivity through the cell membrane of HO_2_ and O_2_NOH at acidic pH are consistent with higher bacterial inactivation efficiency of plasma treatment in acidic pH environment^44^. The CAP treatment induced significant oxidative stress in *Staphylococcus aureus* biofilm bacteria and trigger intracellular production of ROS. The plasma-generated reactive species and plasma induced endogenous ROS possibly contributed to bacteria death synergistically^45.^ The CAP treatment of *Saccharomyces cerevisiae* cells caused oxidative stress responses including the nuclear accumulation of the oxidative stress responsive transcription factor Yap1, mitochondrial fragmentation, and enhanced intracellular oxidation. Yeast cells also induced the expression of heat shock protein (HSP) genes and formation of Hsp104 aggregates when treated with CAP, suggesting that CAP denatures proteins^23,46^. Plasma-generated ROS leads in *S. cerevisiae* to the accumulation of intracellular ROS and Ca^2+^ that ultimately contribute to apoptosis and fragmentation of nuclear DNA^47^.

The CAP treatment of the freshly pressed tomato juice proved effective in inhibition of microbial decontamination, but this effect was clearly dependent on the duration of the treatment.

The investigations have shown that the 30-s CAP treatment of the tomato juice only slightly reduced the microbial count. The total number of aerobic microorganisms in the plasma-exposed juice samples on the individual storage days decreased on average by 0.1–1.4 log_10_ CFU/g. The number of lactic acid bacteria decreased by an average of 0.03–0.2 log_10_ CFU/g, the amount of coliforms was lower by a maximum of 0.1 log_10_ CFU/g, and the amount of yeasts declined by a maximum of 0.2 log_10_ CFU/g. Evidently, the 30-s plasma treatment did not extend the shelf life of the tomato juice substantially.

The 60-s CAP treatment of the tomato juice yielded slightly better results although not fully satisfactory in terms of extending the shelf life of the juice. The comparison of the multiplication of microorganisms in the stored control juice samples with the inhibition of microbial growth in the plasma-treated samples showed the largest reduction in the microflora after 10 days of storage. The total number of aerobic microorganisms decreased by 0.3–2.4 log_10_ CFU/g in samples subjected to the 60-s CAP treatment, compared with the controls. The number of lactic acid bacteria declined by 0.3–2.4 log_10_ CFU/g, whereas the number of coliform bacteria and yeasts was reduced by a maximum of 1.4 log_10_ CFU/g and 1.2 log_10_ CFU/g, respectively.

In comparison with the control, the 120-s CAP treatment yielded the highest reduction in the number of microorganisms after 10 days of storage. The total number of aerobic microorganisms was reduced by 2.3 log_10_ CFU/g after 10 days. This value in the case of both lactic acid bacteria and coliforms was 2.2 log_10_ CFU/g. The yeast cell count declined by 1.2 log_10_ CFU/g after 10 days of storage. The 120-s CAP treatment slightly improved the microbiological purity of the tomato juice samples, in comparison with the control; however, it is difficult to draw definitive conclusions on its effect on the shelf life of the juice.

The 300-s CAP treatment resulted in significant improvement of the microbiological quality of the tomato juice and extended its shelf life to 10 days (in accordance with the allowable total number of aerobic microorganisms and the number of yeasts specified for pasteurized juices by the Regulation of the Polish Minister of Health of January 13, 2003)^41^. The total count of aerobic microorganisms was reduced by 2.4 log_10_ CFU/g after 7 days of storage and by 3.3 log_10_ CFU/g after 10 days in comparison to the control. The number of lactic acid bacteria was reduced by 1.4 log_10_ CFU/g after 7 days of storage and by 5.0 log_10_ CFU/g after 10 days. Regardless of the storage time, the counts of coliform bacteria and yeasts were reduced to < 10 CFU/g (< 1 log_10_ CFU/g), i.e. below the limit of quantification.

The 600-s CAP treatment contributed to extension of the shelf life of the unpasteurized NFC tomato juice to 10 days. This was accompanied by a greater reduction of the microbial count than after the 300-s plasma treatment. The total number of microorganisms was reduced to 1.6 log_10_ CFU/g after 10 days of storage. In turn, the number of lactic acid bacteria, coliforms, and yeasts declined to < 10 CFU/g (< 1 log_10_ CFU/g), which is below the limit of quantification with microbiological methods. After 10 days of storage, the total microbial count and the number of lactic acid bacteria were reduced by 4.2 log_10_ CFU/g and 5.0 log_10_ CFU/g, respectively, compared with the control.

Based on the present results, it can be concluded that CAP generated in the Glidearc device applied for 300–600 s exhibits high potential for decontamination of microorganisms naturally contaminating NFC tomato juice and can be used to extend the shelf life of the product.

Compared to the control, the highest reduction in the number of microorganisms in the cold plasma-treated tomato juice was noted after 10 days of storage. This is related to the multiplication of the microorganisms during storage in the control samples, while their number tended to decline with time in the stored plasma-treated samples.

The phenomenon of gradual inactivation of *Citrobacter freundii* during storage of apple juice treated with CAP has been described by Surowsky et al*.*^30^. The authors observed very low rates of inactivation of this bacterium, i.e. approx. 1 logarithmic cycle after 480-s plasma exposure. In turn, 3-h storage resulted in a 4.4-log reduction. Further extension of the storage time to 24 h resulted in an approx. 5.1-log decrease in the *Citrobacter freundii* count in the analyzed products.

As mentioned earlier, the inactivation of microorganisms in cold plasma-treated biological materials during storage may be associated with the generation of active antimicrobial components e.g. hydrogen peroxide (H_2_O_2_), hydroperoxide radicals (OOH•), and peroxynitrate (III) (ONOO−) as well as their derivatives and other active products of their reactions, which have a longer half-life than OH^•^ and O_2_^•−^ radicals and are active in plasma-treated juice^16,17,30,48^. Additionally, tomato juice has low content of sugars, which are necessary for cells to trigger effective defense mechanisms against oxidative stress induced by active plasma components. The absence of a sufficient energy source, e.g. sugars, can accelerate the death of microbial cells, which are additionally weakened by the action of cold atmospheric plasma.

### Physicochemical analysis

The requirements for raw material and quality of vegetable and fruit-vegetable juices are specified in Polish standard PN-A-75958 *Vegetable and fruit-vegetable products*. *Juices*^49^. The information contained in the standard is imprecise; hence, the market offers an entire range of products with varied quality, mainly their physicochemical characteristics, which to some extent are reflected in their sensory traits. Therefore, any results of analyses of the physical and chemical parameters of juices should be compared with the guidelines of the AIJN^50^ code of practice. Initially, it was formulated to specify the requirements for the quality of fruit juices. Over time, however, due to the increase in the sales of vegetable juices, the code also included data on tomato and carrot juices. Nevertheless, the data available in both documents only indicate that tomato juice should be characterized by pH ≤ 4.4 (PN) and ≤ 4.5 (Code of Practice guidelines). Other quality parameters for tomato juice can be compared only with results reported in scientific publications by other authors in the case of other fruit and vegetables as well as juices produced from these raw materials.

The analysis of the present results indicated that the fresh tomato juice (control sample) immediately after pressing was characterized by the following physicochemical properties: pH – 3.9 ± 0.04; soluble substances – 3.75 ± 0.07^o^Bx; total carotenoids – 77.10 ± 0.57 mg/100 g; vitamin C – 12.68 ± 0.38 mg/100 g.

The effects of CAP on the values of the analyzed parameters are presented in Tables 1, 2, 3 and 4.

**Table 1:** Effect of the action of non-equilibrium low-temperature plasma generated with the use of nitrogen on the acidity of tomato juice stored for 10 days.

Acidity (pH)				
Plasma operating time (s)	Time of storage (days)			
	1	4	7	10
0 (control)	3.99 ± 0.00^Aa^	4.07 ± 0.08^Aab^	4.12 ± 0.10^Ab^	Not marked
30	4.44 ± 0.01^Ba^	4.45 ± 0.01^Ba^	4.46 ± 0.02^Ba^	4.47 ± 0.02^Aa^
60	4.44 ± 0.07^Ba^	4.45 ± 0.01^Ba^	4.47 ± 0.00^Bb^	4.48 ± 0.01^Ab^
120	4.46 ± 0.00^Ba^	4.46 ± 0.00^Ba^	4.49 ± 0.02^Bb^	4.49 ± 0.01^Ab^
300	4.47 ± 0.02^Ba^	4.48 ± 0.00^Ba^	4.47 ± 0.07^Bb^	4.51 ± 0.02^Ab^
600	4.47 ± 0.00^Ba^	4.49 ± 0.00^Ba^	4.94 ± 0.07^Cb^	4.90 ± 0.03^Bb^

The results are expressed as a mean ± standard error.

Average values in the column marked with the same capital letter are not statistically significantly different (p < 0.05).

Average values in the raw small marked with the same small letter are not statistically significantly different (p < 0.05).

**Table 2:** Effect of the action of non-equilibrium low-temperature plasma generated with the use of nitrogen on the soluble substances of tomato juice stored for 10 days.

Soluble substances (^o^Brix)				
Plasma operating time (s)	Time of storage (days)			
	1	4	7	10
0 (control)	3.65 ± 0.07^Aa^	3.55 ± 0.07^Ba^	3.50 ± 0.14^Ca^	not marked
30	3.75 ± 0.07^Aa^	3.85 ± 0.07^ABa^	3.90 ± 0.00^Aa^	4.05 ± 0.07^Aa^
60	3.80 ± 0.00^ABa^	3.90 ± 0.00^Aa^	3.95 ± 0.07^ABa^	4.00 ± 0.14^Aa^
120	3.85 ± 0.07^ABa^	3.95 ± 0.07^Aa^	4.05 ± 0.07^ABa^	4.00 ± 0.14^Aa^
300	3.90 ± 0.00^ABa^	3.95 ± 0.07^Aa^	4.05 ± 0.07^ABa^	3.95 ± 0.07^Aa^
600	4.10 ± 0.14^Ba^	4.10 ± 0.14^Aa^	4.25 ± 0.07^Ba^	4.10 ± 0.14^Ab^

The results are expressed as a mean ± standard error.

Average values in the column marked with the same capital letter are not statistically significantly different (p < 0.05).

Average values in the raw small marked with the same small letter are not statistically significantly different (p < 0.05).

**Table 3:** Effect of the action of non-equilibrium low-temperature plasma generated with the use of nitrogen on the carotenoids of tomato juice stored for 10 days.

Carotenoids (mg/100 g)				
Plasma operating time (s)	Time of storage (days)			
	1	4	7	10
0 (control)	77.50 ± 0.71^Ab^	76.50 ± 0.57^Ab^	72.55 ± 0.07^Aa^	Not marked
30	77.60 ± 0.42^Aa^	77.05 ± 1.34^Aa^	75.25 ± 2.76^ABa^	74.25 ± 1.91^Aa^
60	77.35 ± 0.78^Aa^	77.20 ± 0.85^Aa^	76.10 ± 0.57^ABa^	75.65 ± 1.06^Aa^
120	77.45 ± 0.35^Aa^	77.25 ± 0.21^Aa^	76.70 ± 0.42^ABa^	76.05 ± 0.49^Aa^
300	78.00 ± 0.00^Aa^	78.05 ± 0.21^Aa^	77.40 ± 0.42^Ba^	76.35 ± 1.48^Aa^
600	79.15 ± 0.49^Ab^	79.15 ± 0.49^Aab^	77.10 ± 0.14^ABab^	74.95 ± 2.47^Aa^

The results are expressed as a mean ± standard error.

Average values in the column marked with the same capital letter are not statistically significantly different (p < 0.05).

Average values in the raw small marked with the same small letter are not statistically significantly different (p < 0.05).

**Table 4:** Effect of the action of non-equilibrium low-temperature plasma generated with the use of nitrogen on the ascorbic acid of tomato juice stored for 10 days.

Ascorbic acid (mg/100 g)				
Plasma operating time (s)	Time of storage (days)			
	1	4	7	10
0 (control)	12.27 ± 0.04^Ac^	11.70 ± 0.02^Bb^	9.09 ± 0.01^Ba^	Not marked
30	12.21 ± 0.13^Aa^	12.25 ± 0.06^Aa^	12.13 ± 0.02^Aa^	12.15 ± 0.03^Aa^
60	12.21 ± 0.08^Aa^	12.23 ± 002^Aa^	12.21 ± 0.01^ Da^	12.12 ± 0.03^Aa^
120	12.12 ± 0.03^Aa^	12.12 ± 0.02^ACa^	12.10 ± 0.01^Aa^	12.02 ± 0.03^ABa^
300	12.10 ± 0.03^Ab^	12.04 ± 0.04^Cab^	12.08 ± 0.01^Aab^	11.94 ± 0.06^Bb^
600	11.75 ± 0.06^Ba^	11.69 ± 0.03^Ba^	11.70 ± 0.01^Ca^	11.70 ± 0.01^Ca^

The results are expressed as a mean ± standard error.

Average values in the column marked with the same capital letter are not statistically significantly different (p < 0.05).

Average values in the raw small marked with the same small letter are not statistically significantly different (p < 0.05).

In general, pH has a considerable impact on the nutritional value or shelf life of fruit and vegetable juices. Changes in acidity can provide information about adverse processes that may occur in products (e.g. resulting from improper processing or inadequate storage), freshness, and even toxicity. Furthermore, the acidity of juices often determines the method employed in the technological process or qualification of the raw material for the type of production. The pH value determined in the control juice samples after 24-h storage was 3.99. The cold plasma-treated samples exhibited a slight increase in pH throughout the storage time. In turn, the 7- and 10-day refrigeration storage of the raw material subjected to the 60- to 600-s treatments induced changes in the analyzed parameter in comparison with samples analyzed after 24 h (Table 1).

The pH of the tomato juice in the present study corresponds to the values reported by Dasan et al*.*^28^, who subjected tomato juice to atmospheric pressure plasma and found that the pH values of the plasma-treated samples did not differ from those of the control sample (4.31). The authors observed a slight increase in this parameter only at the processing time of 90 s (4.33) and 120 s (4.33). In investigations conducted by Pankaj et al*.*^31^, grape juice samples were processed at 80 kV for 1, 2, 3, and 4 min. The pH of plasma-untreated material was 3.38, and there was no significant difference in the parameter after the atmospheric plasma treatment. Misra et al.^51^ demonstrated an increase in the pH of control cherry tomatoes at the end of the storage period and in tomatoes subjected to 30-, 60-, 180-, and 300-s plasma treatment. The increase in pH was slightly higher in the plasma-treated tomatoes and inversely proportional to the processing time. However, there was no statistically significant difference (p < 0.05) between the pH value of the control sample and those subjected to cold atmospheric plasma. In their experiments, the authors applied cold air plasma generated inside a bag using a dielectric barrier. However, there are literature reports showing a decrease in pH values in freshly pressed orange juice after plasma treatment^52^. Almeida et al*.*^33^ generated plasma with a dielectric barrier discharge (at 70 kV in atmospheric air and at atmospheric pressure conditions). The authors of the study found that the pH of prebiotic orange juice samples treated with cold atmospheric plasma was statistically different from the value determined in the control sample. The latter had a mean pH value of 4.43, whereas the pH of the plasma-treated orange juice samples ranged from 3.9 to 4.0. The differences in the pH of tomatoes and their products are attributed to metabolic changes and water loss in the processed material^53^. Decrease in acidity could be due to the solubilization of hydroxyl radicals generated during the plasma discharge^31^. On the other hand radicals (for example, OH·) or ozone generated by plasma could oxidize part of aldehydes in juice into acid compounds. The reactive nitrogen species RNS formed during plasma treatment could produce nitrogenous acids, resulting in increased acidity of material^27^.

°Brix is an important index for determination of the quality of juices. The content of soluble substances in the control tomato juice on the first day of storage was determined at a level of 3.65^o^Brix. There were no statistically significant differences between these samples and those exposed to cold atmospheric plasma (except for the 600-s treatment). The values of soluble substances were in the range of 3.75–4.10 ^o^Brix. The storage time had no significant effect on this parameter (Table 2).

Liao et al*.*^27^ did not observe significant differences (P > 0.05) in the °Brix value for untreated and DBD‐ACP‐treated (the input power intensity was set at 30, 40, and 50 W, and the treatment times were 0 to 40 s) samples of apple juice. The findings were in accordance with the observation of Shi et al.^52^ and Xu et al*.*^22^ who found that a dielectric barrier discharge plasma treatment and high voltage atmospheric cold plasma HVACP had no significant effect on the °Brix of orange juices. According to Wang et al.^54^ the slight increase in °Brix could be attributed to the loss of moisture during ACP treatment.

Carotenoids are responsible for the yellow to red color of plants. They are efficient antioxidants involved in scavenging singlet oxygen and peroxide radicals. In the human organism, carotenoid antioxidants are part of the defense system against free radicals.

The cold plasma treatment contributed to a slight increase in the total carotenoid content in the tomato juice, compared to the control (77.50 mg/100 g). The maximum content of these compounds was detected on storage days 1 and 4 in samples treated with cold plasma for 600 s (79.15 mg/100 g). During storage, the cold plasma-treated juice samples exhibited a slightly higher carotenoid level than the control. However, a statistically significant difference was noted only after 7 days in the case of juice exposed to the 300-s treatment. The lowest value of these compounds, i.e. 74.25 mg/100 g, was observed in tomato juice treated with cold plasma for 30 s after 10 days of storage (Table 3).

In their analysis of the content of carotenoids in kiwi fruit, Ramazzina et al.^55^ found that the storage time reduced the amount of these compounds in the fruit; however, the pigments were more stable in plasma-exposed samples than in the control. It can be assumed that degradation of carotenoids is influenced by cold atmospheric plasma and oxidizing compounds formed in this process as well as a prolonged storage time. In the study conducted by Paixão et al*.*^56^, the use of a 10 and 20 mL/min flow rate of nitrogen for 5–15 min resulted in an increase in the relative percentage of carotenoid compounds in siriguela juice (109.47–126.69%) relative to the control (100%). A gas flow rate of 30 mL/min was responsible for reduction of the content of these pigments, irrespective of the processing time (86.41–92.73%). Nitrogen glow plasma was found to increase the content of carotenoids in acerola juice in specific processing conditions. The highest increase in the content of these substances was recorded at a gas flow rate of 10 mL/min and 10 min of treatment time^57^.

Tomato juices are a rich source of compounds with antioxidant properties, primarily vitamin C. It has the ability to destroy lipid peroxides and scavenge free radicals generated during food preparation and via metabolic processes in the organism. Unfortunately, vitamin C is heat-labile and easily destabilized during thermal processing or post-storage. Therefore, vitamin C retention is an indicator of the processing severity^58,59^.

The content of vitamin C in the control tomato juice (on storage day 1) was 12.27 mg/100 g. The 600-s cold plasma decontamination caused only a slight loss of vitamin C (maximum 5%). The reduction of the ascorbic acid content during the cold plasma treatment may have been associated with oxidation reactions supported by the interaction of free radicals and ozone generated during this process. The juice storage time did not exert a significant impact on the content of ascorbic acid in the analyzed samples (Table 4).

With regard to orange juice, the concentration of vitamin C decreased by 22% (in air) after 120-s HVACP direct treatment in 25-mL air packed product compared with untreated sample^22^. This loss of vitamin C may arise due to the high concentration of ROS and RNS in the reactive gas species generated by HVACP. Ascorbic acid is sensitive to O_3_ and easily degraded by other free radicals^60,61^. The application of indirect plasma (using N_2_ flow rates of 10, 30 and 50 mL/min during 5, 10 and 15 min) on the cashew apple by Rodríguez et al*.*^32^ promoted significant changes in the vitamin C content. When a N_2_ flow rate of 10 mL/min was used, an increment of 10.4 and 10.8% was observed after 5 and 10 min of treatment, respectively, but when the treatment was extended to 15 min the sample exhibited a reduction of 4.5% of those substances. Paixão et al.^56^ also noted that there was no difference between siriguela juice samples after the glow discharge plasma processing with nitrogen for 5, 10 or 15 min at 10, 20 and 30 mL/min (13.70–16.66 mg/100 mL) and the control sample (15.18 mg/100 mL).

### Microstructure analysis

**Figure 3:**
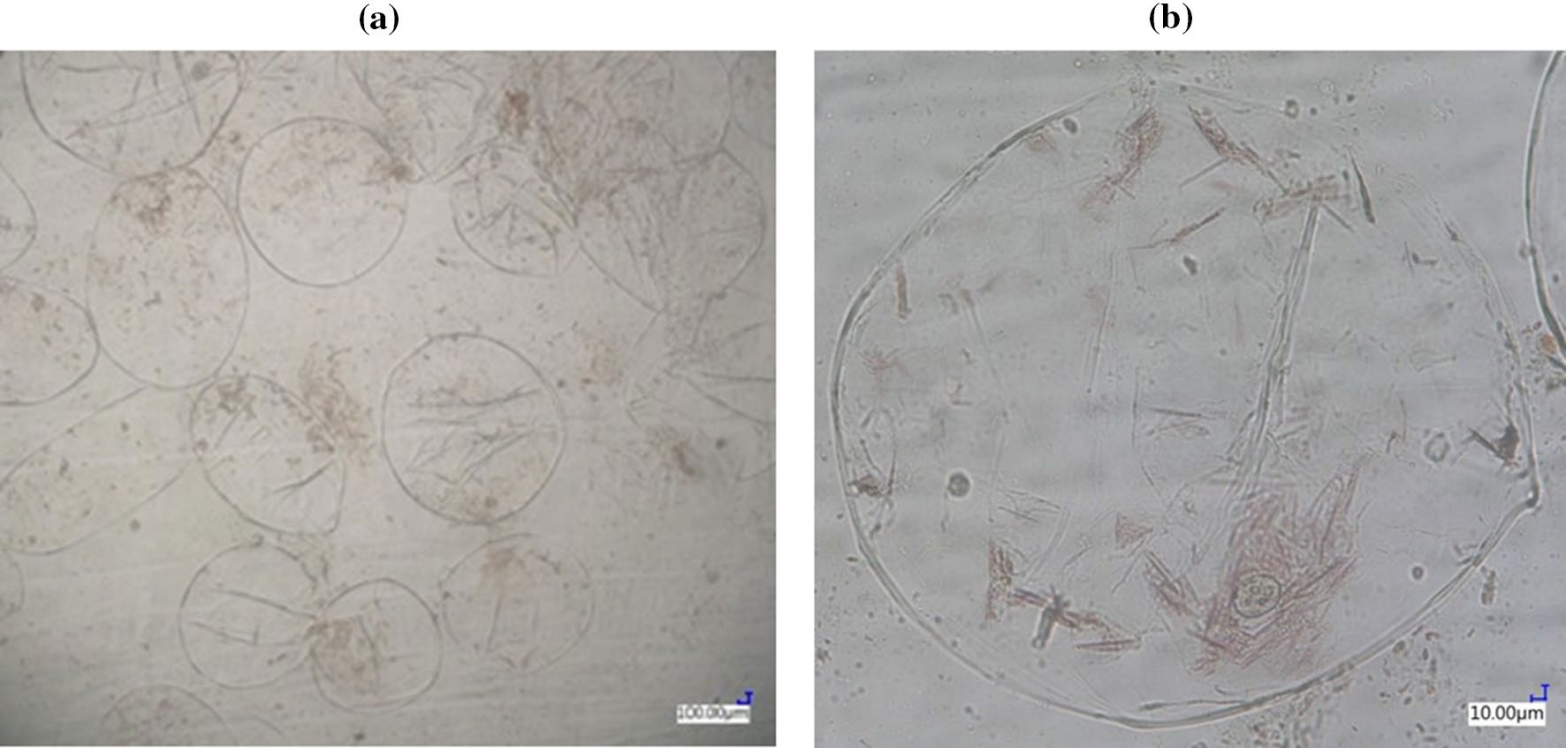
Microstructure of fresh NFC tomato juice enlarged **(a)** × 100 and **(b)** × 500.

The fresh tomato juice (control sample) contained undamaged cells with intact chromoplasts, i.e. organelles containing carotenoids, including lycopene, which are visible as red “needles” called lycopene crystals (Fig. 3).

Most of them are relatively soft parenchyma cells from the mesocarp, since the tomato skin and seeds were removed during juice production.

**Figure 4:**
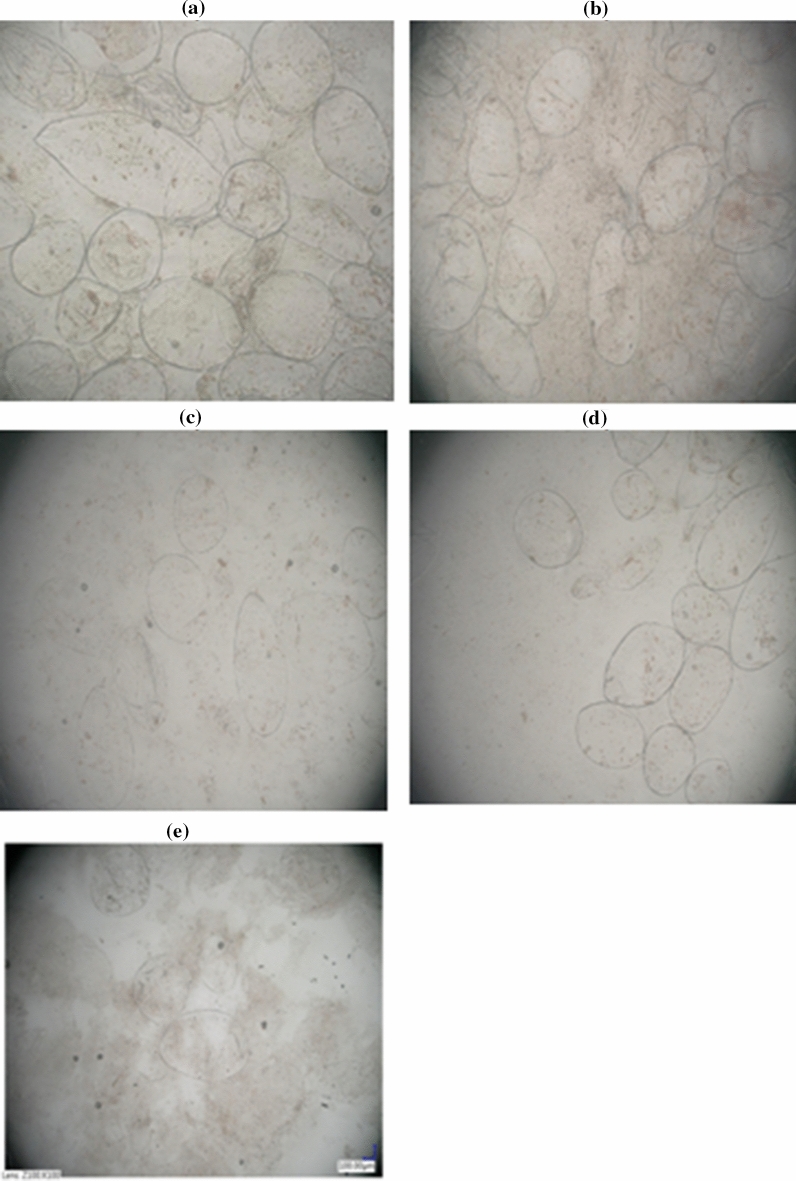
The microstructure of tomato juice (× 100) exposed to cold atmospheric plasma for: **(a)** 30 s, **(b)** 60 s, **(c)** 120 s, **(d)** 300 s and **(e)** 600 s.

Figure 4 shows digital microscope images of tomato juice exposed to cold plasma. The product subjected to the 30-, 60-, and 120-s treatment contained clusters of several cells (with lycopene crystals visible inside intact chromoplasts). In contrast, the material subjected to the treatment for 300 and 600 s consisted of whole cells suspended in juice homogenate and cells with visible amorphous clusters of the red pigment, which indicates only slight damage to the chromoplasts. To our knowledge, the microscopic images presented in this study are the first data of the microstructure of NFC tomato juice exposed to cold atmospheric plasma.

## Conclusions

Cold atmospheric plasma generated with the use of nitrogen in a Glide-arc device for 300 to 600 s exhibited high potential for decontamination of microorganisms and did not cause simultaneous changes in the physicochemical properties of tomato juice. The 300-s treatment resulted in significant improvement of the microbiological quality of the juice and extension of its shelf life to 10 days. The prolonged cold plasma treatment time (600 s) led to further reduction in the number of microorganisms, in comparison with the 300-s treatment. The total count of microorganisms, i.e. lactic acid bacteria, coliform bacteria, and yeast, were reduced below the limit of quantification. The CAP-exposed samples of tomato juice exhibited an intact structure, as revealed by digital microscopy. The study suggests that CAP can be successfully implemented on an industrial scale for processing NFC tomato juice.
